# Recipient rs1045642 Polymorphism Is Associated With Office Blood Pressure at 1-Year Post Kidney Transplantation: A Single Center Pharmacogenetic Cohort Pilot Study

**DOI:** 10.3389/fphar.2018.00184

**Published:** 2018-03-05

**Authors:** Yassine Bouatou, Ludwig Stenz, Belen Ponte, Serge Ferrari, Ariane Paoloni-Giacobino, Karine Hadaya

**Affiliations:** ^1^Division of Clinical Pharmacology and Toxicology, Geneva University Hospitals, Geneva, Switzerland; ^2^Division of Nephrology, Geneva University Hospitals, Geneva, Switzerland; ^3^Department of Genetics Medicine and Development, Geneva University, Geneva, Switzerland; ^4^Division of Bone Diseases, Geneva University Hospitals, Geneva, Switzerland; ^5^Division of Transplantation, Geneva University Hospitals, Geneva, Switzerland

**Keywords:** rs1045642, C3435T, kidney, transplantation, diastolic, systolic, blood pressure

## Abstract

**Background:** Corticosteroids are associated with reduced bone mineral density (BMD), as well as water and salt retention, leading to hypertension. They are substrates for P-glycoprotein, a protein coded by the highly polymorphic *ABCB1* gene. We hypothesized that one *ABCB1* polymorphism, rs1045642, is associated with blood pressure and BMD parameters at 1-year post kidney transplantation (KT).

**Methods:** Rs1045642 was genotyped using pyrosequencing in 40 KT recipients. Both dominant (CC vs. CT + TT) and codominant (CC vs. CT vs. TT) genetic models (analysis of variance from linear regressions) were adjusted for confounding variables (age, sex, type of nephropathy, glomerular filtration rate, and corticosteroid use at 1 year).

**Results:** Rs1045642 genotypes were significantly associated with systolic (SBP) and diastolic (DBP) blood pressure 1-year post-transplantation, independent of the genetic model used (adjusted codominant model: SBP *p*-value = 0.015, DBP *p*-value = 0.038; adjusted dominant model: SBP *p*-value = 0.003, DBP *p*-value = 0.011). A non-statistically significant trend was observed for an association between rs1045642 and BMD change at 1-year post-KT.

**Conclusions:** Rs1045642 is significantly associated with higher BP 1 year after KT. Further investigations are necessary to confirm the role of rs1045642 in corticosteroid-related adverse effects.

## Introduction

Corticosteroids continue to be used after kidney transplantation (KT) in immunosuppression protocols worldwide. Among the known adverse effects associated with prednisone use, dyslipidemia, diabetes, hypertension, and cataracts have been well evaluated in contexts other than KT (Bergmann et al., 2012).

Elevated systolic blood pressure (SBP) and diastolic blood pressure (DBP) are risk factors for graft failure and cardiovascular disease after KT (Opelz et al., 1998). Hypertension is frequently encountered in KT recipients (Opelz et al., 1998). A number of risk factors for high BP after KT have been identified, including preexisting hypertension, use of calcineurin inhibitors and corticosteroid, poor donor organ quality, and an increased body mass index. Substantial loss of bone mass has also been reported during the first year after KT, with corticosteroids being identified as a key contributor to this phenotype (Dounousi et al., 2015). Accordingly, many transplant centers now add a bisphosphonate to their post-KT protocols to prevent osteoporosis (Wang et al., 2016). However, interindividual variability exists regarding the propensity of corticosteroids to induce hypertension or osteopenia. Understanding the factors associated with this variability will help clinicians stratify patients according to their risk of developing these adverse events and, thereby, adjust immunosuppressive protocols accordingly.

P-GP, encoded by the *ABCB1* gene, is a member of the superfamily of ATP-binding cassette (ABC) transporters. ABC proteins transport several molecules across extra- and intracellular membranes. More specifically, P-GP is a member of the multidrug resistance (MDR) family known as the MDR/TAP subfamily (Hodges et al., 2011). P-GP acts as an ATP-dependent drug efflux pump with broad substrate specificity for endogenous substances and xenobiotics, including prednisone and prednisolone, and is expressed on the membranes of various cells (Hodges et al., 2011), such as lymphocytes and hepatocytes.

Prednisone is a substrate and an inducer of the polymorphic P-GP (Crowe and Tan, 2012; Manceau et al., 2012). Among the single nucleotide polymorphism (SNP) in *ABCB1*, the synonymous rs1045642 (C3435T) affects the P-GP protein either by being in linkage disequilibrium with others functional SNPs, or by allele-specific differences in the codon usage affecting the protein folding and changing the substrate specificity (Kimchi-Sarfaty et al., 2007). Interestingly, the association of *ABCB1* with blood pressure or, bone mineral density (BMD) in the context of corticosteroids, has been previously evaluated (Bochud et al., 2009; Løvås et al., 2009).

Our study aims to identify the association between *ABCB1* polymorphisms and blood pressure, as well as change in bone mineralization [represented by t-score and (BMD) measurements], at 1-year post-KT. We hypothesize that rs1045642 (C allele) is associated with higher blood pressure and increased loss of BMD at 1 year after transplantation.

## Materials and methods

### Study design

This pharmacogenetics, cohort pilot study is a single center, monogenic association study evaluating the relationship between the SNP rs1045642 and corticosteroid-induced adverse events, defined as blood pressure and changes in bone mineralization phenotypes at 1 year, among KT recipients.

The primary outcomes were SBP and DBP measurements at 1 year post-KT. Secondary outcomes were changes in BMD and t-score at 1-year post-KT. The methods and results are reported according to STrengthening the REporting of Genetic Association Studies (STREGA), an extension of the STROBE Statement (Little et al., 2009).

### Ethics statement

The study was approved by the Geneva ethical commission under study number CER-14-243. All patients included in the study voluntarily provided informed and written consent during the recruiting process, in accordance with the principles of both the Declaration of Helsinki and the “Declaration of Istanbul on Organ Trafficking and Transplant Tourism.”

### Participants

The inclusion criteria were as follows: single organ transplantation; age >18 years old on the transplantation day; Caucasian ethnicity; first kidney allograft transplanted between January 2005 and September 2014 at the Geneva University Hospitals; dual-energy x-ray absorptiometry (DXA) in the week following transplantation and at 1 year post-KT; and informed and written consent provided for the genetic and retrospective medical record analyses. The exclusion criteria were as follows: non-Caucasian ethnicity; post-KT protocol not involving the use of a corticosteroid; corticosteroid use during the 12 months before transplantation; or treatment for a humoral or tubulointerstitial rejection episode during the first post-KT year.

### Variables

The following outcomes were defined and used as outcome variables: SBP and DBP at 1-year post-KT; BMD change at 1-year post-KT, defined as the difference between the 1-year BMD (g/cm^2^) and the baseline BMD (g/cm^2^); and t-score change at 1-year post-KT, defined as the difference between the 1-year t-score and the baseline t-score. These outcomes were all continuous variables.

The following covariables were also evaluated: age at the time of transplantation (years); sex; glomerular filtration rate (eGFR; mL/min/1.73 m^2^), estimated from the creatinine-based formula developed by the Chronic Kidney Disease Epidemiology Collaboration (Levey et al., 2009); corticosteroid use 1 year after transplantation [defined as a categorical variable (yes or no)]; and use of bisphosphonate and proton pump inhibitors (PPIs) at 1-year post-KT [defined as categorical variables (yes or no)]. The glomerular filtration rate was calculated using the formula from the Chronic Kidney Disease Epidemiology Collaboration: GFR = 141 × min(Scr/κ, 1)^α^ × max(Scr/κ, 1)^−1.209^ × 0.993^Age^ × 1.018 [if female] × 1.159 [if black]. Scr is serum creatinine in μmol/L; κ is 61.9 for females and 79.6 for males; α is −0.329 for females and −0.411 for males; min indicates the minimum of Scr/κ or 1, and max indicates the maximum of Scr/κ or 1. The equation does not require weight because the results are reported normalized to 1.73 m^2^ body surface area, which is an accepted average adult surface area.

In our standard post-KT protocol, corticosteroids were progressively tapered at 3 months until the 6 month post-KT if the following were applicable: rejection was not present within the first 3 months; a daily mycophenolate mofetil dose more than 1.5 g per day was able to be sustained; and the patient did not have a preexisting nephropathy for which there was a risk of recurrence. Otherwise, the patients were maintained on a prednisone 5 mg per day regimen. Bisphosphonates were administrated to patients presenting an osteopenia or an osteoporosis at baseline.

Information about antihypertensive regimen was extracted from the electronic medical records. We looked for any antihypertensive drugs in the patient's treatment list at admission for transplantation and at 1 year post kidney transplantation such as: diuretics, calcium channel antagonists, beta-blockers, central blockers, angiotensin converting enzyme inhibitors or angiotensin receptor antagonists or renin inhibitors. We reported and compared the number of drugs before and after the transplant. Hypertension was defined as the presence of at least one antihypertensive drug.

### Data sources and measurements

#### Blood pressure measurements

Office SBP and DBP were determined by a single measurement obtained with the patient in the sitting position after resting for 15 min. The BP was measured using a calibrated non-mercury sphygmomanometer (Royal Philips Electronics, the Netherlands) that complied with the standards from the British Hypertension Society protocol for the evaluation of blood pressure measuring devices (O'Brien et al., 1990).

#### BMD measurements

Lumbar spine, right femoral neck, and proximal right hip BMD values (expressed in g/cm^2^) and the corresponding t-scores were measured during the first week post-transplantation (t0) and at 1-year post-transplantation (t1) by DXA using bone densitometers (QDR-4500 and Discovery A; Hologic, Inc, Bedford, MA, USA) with a coefficient of variation of repeated measurements varying between 1.0 and 1.5%, as previously reported (Chevalley et al., 2005a,b; Calmy et al., 2013).

#### DNA extraction and RS1045642 genotyping

DNA was extracted using the Gentra® Puregene Blood kit (Qiagen, Germany) according to the manufacturer's instructions from 3-mL blood samples collected in ethylenediamine tetra-acetic acid–coated tubes. RS1045642 was determined by pyrosequencing using an assay developed for this study. Primers were designed with Primer3 (Untergasser et al., 2012), resulting in the forward primer “F_RS1045642” 5′-TTAGGCAGTGACTCGATGAAGG-3′ and the reverse primer “R_RS1045642” 5′-AGCTGCTTGATGGCAAAGA-3′, which was biotinylated at the 5′ end. The polymerase chain reaction (PCR) reaction mixture included 12.5 μL HotStarTaq® Master Mix, 0.5 μL F_RS1045642, 0.5 μL R_RS1045642, 10.5 μL water, and 1 μL DNA (100 ng/μL). Amplifications were performed in a Veriflex Thermocycler (Applied Biosystems, USA) using these parameters: 95°C for 15 min; followed by 50 cycles of 94°C for 30 s, 62°C for 30 s, and 72°C for 30 s; and final extension at 72°C for 1 min. The PCR products were subjected to strand separation using Streptavidin Sepharose™ High Performance beads (GE Healthcare, United Kingdom) in a PyroMark Q24 vacuum workstation, then sequenced using the sequencing oligo “S_RS1045642” 5′-CTCCTTTGCTGCCCT-3′ in a PyroMark™ Q24 Instrument (Qiagen) using appropriate enzymes and substrate mixtures, as well as nucleotides (PyroMark™ Gold Reagents, Qiagen). RS1045642 genotypes were determined automatically by the instruments and each pyro-histogram was verified. The sequence to analyze was CAC**R**ATCTCTTCCTGTGWCACCACCCGGCTGTTGTCTCCATARGCAATGTTCTCAGCAATGCTGCAGTCAAACAGGATGGGCTCCTGGGACACGATGCCCAGGTGTGCTCGGAGCCRCTGAACATTCAGTCGCTTTATTTCT, and the dispensation order was ACATCGATCTCTCTGTCGATCACACGCTGTGTCTCAGTAGCATGTCTCAGCATGCTGCAGTCACAGATGCTCTGACACGATGCAGTGTGCTCGAGTCAGCTG. RS1045642 was the genotype on the reverse strand at the bold R position in the sequence to analyze, with R = A or G corresponding to Y = T or C, respectively, on the complementary strand; this resulted in three possible genotypes: CC (or GG), CT (or GA), and TT (or AA).

### Study size

Frequency of the C allele has been reported to vary between 43 and 48% among Caucasians (Ameyaw et al., 2001). Sample size calculations indicated that 15 CC and 15 CT or TT patients were required to detect a 15 mm Hg difference in SBP between groups (a difference we considered to be clinically relevant), with a standard deviation estimated at 14 mm Hg, a significance threshold α of 0.05, and a power of 80% (http://www.openepi.com/).

### Statistical methods

All statistical analyses were performed using Stata version 14.1 and RStudio package version 3.0.2. Departure from the Hardy–Weinberg equilibrium for the three genotype groups (CC, CT, and TT) was tested in Stata 14.1 using a chi-square test (*hwsnp* function) and was found to be not significant. The 95% confidence intervals were calculated using Rmisc (https://cran.r-project.org/web/packages/Rmisc/Rmisc.pdf). Analysis of variance (ANOVA) for linear model fits were performed in R using lm and ANOVA functions for both univariate and multivariate analyses (Chambers and Hastie, 1992). Briefly, linear model fits in function of the genotypes corresponding to linear regression were performed separately for each of the following outcomes: SBP, DBP, and six measurements of bone mineralization (BMD_evol 1, 2, and 3 and T_evol 1, 2, and 3, representing BMD and t-score values at lumbar spine, right femoral neck, and proximal right hip, respectively). The codominant model compared all three possible genotypes separately, whereas the dominant model compared CT and TT grouped together vs. CC. For the models evaluating SBP and DBP outcomes, the eGFR at 1-year post-KT, age, sex, type of nephropathy, and corticosteroid use at 1 year were used as covariates. For the models evaluating BMD-associated outcomes, age, sex, corticosteroid use at 1 year, bisphosphonate use at 1 year, and PPI use at 1 year were considered as confounders. Following this, ANOVA tables were computed from the linear model fits. A *p*-value < 0.05 was considered statistically significant.

## Results

In this study, we included 40 patients who provided informed and written consent and fulfilled the inclusion and exclusion criteria. The patients' main baseline characteristics are presented in Table 1 according to their rs1045642 genotype. The study included mostly males (72.5%), and the mean age of all patients was 52.5 years. No relevant differences were found between groups in terms of baseline characteristics. Of note, no differences were observed among genotypes in terms of cold ischemia time (*p* = 0.13). Regarding hypertension treatment, there were no differences according to genotypes in the number of antihypertensive drugs taken, before transplantation (*p* = 0.98) and at 1 year post transplantation (*p* = 0.65). There were also no statistically significant differences among genotype groups in the proportion of patients with rejection diagnosed at last follow-up, the proportion taking tacrolimus or cyclosporine at 1 year, or the trough levels of tacrolimus or cyclosporine at 1 year (data not shown).

**Table 1:** Baseline patient characteristics by genotype (*n* = 40).

	**CC (*n* = 15)**	**CT (*n* = 18)**	**TT (*n* = 7)**
Age at transplantation, mean (*SD*)	53 (16)	52 (13)	52 (16)
Sex, *n* of male (%)	10 (66)	13 (72)	6 (85)
Height in cm, mean (*SD*)	167.1 (11.3)	170.7 (9.8)	170.7 (5.1)
Weight in kg, mean (*SD*)	73.3 (18.1)	78.9 (14.9)	76.0 (11.2)
BMI, mean (*SD*)	25.6 (5.2)	27 (4.4)	26.2 (4.6)
Type of Nephropathy, *n* (%)			
Diabetic nephropathy	1 (6)	2 (11.1)	1 (14.3)
Hypertension	3 (20)	1 (5.5)	1 (14.3)
Glomerulonephritis	6 (40)	10 (55.5)	1 (14.3)
Other/Unknown	5 (33.3)	5 (27.7)	4 (57.1)
Previous dialysis, *n* (%)	12 (80)	15 (83.3)	6 (85.7)
Cold ischemia time, mean (*SD*)	431 (398)	644 (488)	268 (365)
Hypertension before transplantation, *n* (%)	13 (86.6)	15 (83.3)	5 (71.4)
Antihypertensive drugs number before transplantation, mean (*SD*)	2.0 (1.4)	2.0 (1.4)	2.1 (2.1)
Hypertension at 1-year post transplantation, *n* (%)	12 (80.0)	14 (77.7)	4 (57.1)
Antihypertensive drugs number1-year post transplantation, mean (*SD*)	1.5 (1.4)	1.9 (1.5)	1.4 (1.6)
Lumbar spine BMD in g/cm^2^, mean (*SD*)	0.977 (0.156)	1.023 (0.131)	1.067 (0.205)
Lumbar spine t-score (*SD*)	−0.9 (1.5)	−0.4 (1.1)	−0.1 (1.9)
Proximal right hip BMD in g/cm^2^, mean (*SD*)	0.864 (0.207)	0.908 (0.140)	0.0941 (0.167)
Proximal right hip t-score (*SD*)	0.7 (0.2)	0.7 (0.1)	0.7 (0.1)
Femoral neck right hip BMD in g/cm^2^, mean (*SD*)	0.732 (0.198)	0.759 (0.137)	0.760 (0.135)
Femoral neck right hip t-score (*SD*)	−1.3 (1.6)	−1.2 (1.0)	−1.2 (1.0)
Use of bisphosphonate after kidney transplantation, *n* (%)	10 (66.6)	13 (72.2)	5 (71.4)

*SD, standard deviation; n, number; BMI, body mass index; BMD, bone mineral density*.

**Figure 1:**
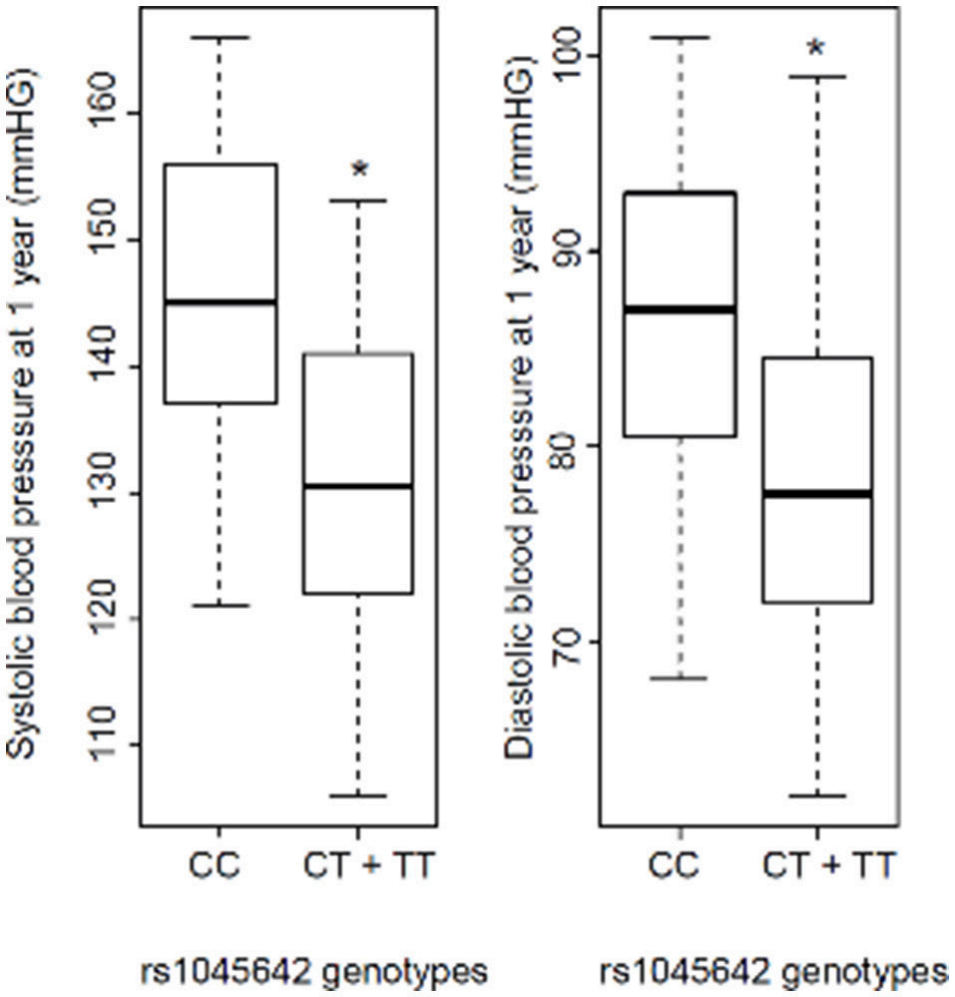
Systolic blood pressure (SBP) and diastolic blood pressure (DBP) 1 year after kidney transplantation according to rs1045642 genotype (CC vs. CT + TT). ^*^p = 0.003 and 0.01 for SBP and DBP, respectively (Mann–Whitney test).

BP results showed a 14.65 mmHg SBP difference between the CC and CT + TT groups. Linear regression analysis was performed for predicting SBP based on rs1045642 genotype. A significant regression equation was generated [*F*_(2 degrees of freedom, 36 residual)_ = 5, *p* = 0.012], with an *R*^2^ of 0.2. Similarly, significantly higher SBP and DBP values (reaching statistical significance) were identified at 1 year among KT recipients carrying the C allele according to the codominant model, as well as in the CC genotype when using the dominant model (CC vs. CT + TT; Table 2). This was observed for both SBP and DBP in univariate analysis and confirmed in multivariate analysis. Statistically significant higher mean SBP and DBP values were observed in CC patients (145/86 mm Hg) vs. CT + TT patients (130/78 mm Hg; Figure 1).

**Table 2:** Association of *ABCB1* polymorphism (rs1045642; CC vs. CT + TT) with systolic and diastolic blood pressure 1-year after kidney transplantation.

**Blood pressure**	**Genotype**			**Codominant model: CC vs. CT vs. TT**		**Dominant model: CC vs. CT** + **TT**	
	**CC**	**CT**	**TT**	**Univariate**	**Multivariate**	**Univariate**	**Multivariate**
	**Mean [95% CI]**	**Mean [95% CI]**	**Mean [95% CI]**	***R***^2^**; F (Df, residual);** ***p*****-value**	***R***^2^**; F (Df, residual);** ***p*****-value**	***R***^2^**; F (Df, residual);** ***p*****-value**	***R***^2^**; F (Df, residual);** ***p*****-value**
SBP	145.1 [137.4, 152.7]	130.7 [123.6, 137.7]	129.7 [114.6, 144.8]	**0.2; 5.0 (2, 36); 0.012**	**0.3; 4.9 (2, 30); 0.015**	**0.2; 10.3 (1, 37); 0.003**	**0.3; 10 (1, 31); 0.004**
DBP	86.5 [81.3, 91.7]	78.1 [73.3, 83.0]	78.7 [67.1, 90.2]	**0.2; 3.3 (2, 36); 0.048**	**0.4; 3.6 (2, 30); 0.038**	**0.2; 6.78 (1, 37); 0.013**	**0.4; 7.4 (1, 31); 0.011**

*CI, confidence interval; SBP, systolic blood pressure; DBP, diastolic blood pressure; Df, degrees of freedom. R^2^ refers to the ratio of explained variation over total variation, varying between 0 and 1. F refers to the model mean square over the residual mean square. Residual refers to the difference between the observed value of the outcome variable and the predicted value. P-value refers to the probability of obtaining a result equal to or more extreme than the result that was obtained, assuming that the null hypothesis is true. The multivariate analyses involved adjustments for the quantitative covariables age at transplantation and estimated glomerular filtration rate at 1-year post-transplantation, as well as the categorical covariables sex, type of nephropathy, and presence or absence of prednisone treatment at 1-year post-transplantation. Significant associations appeared in bold*.

When examining BMD and t-score evolution from baseline to 1-year post-KT, we observed trends reflecting mineral gain in the presence of the T allele and stability of the BMD and t-scores in the presence of the C allele (Supplemental Figure 1). The trends were not statistically significant (Table 3); however, these findings are clinically relevant in this population at high risk of bone demineralization.

**Table 3:** Association of *ABCB1* polymorphism (rs1045642; CC vs. CT + TT) with bone mineralization changes at 1 year after kidney transplantation.

**Bone mineral density values and t-scores**	**Genotype**			**Codominant model: CC vs. CT vs. TT**		**Dominant model: CC vs. CT** + **TT**	
	**CC**	**CT**	**TT**	**Univariate**	**Multivariate**	**Univariate**	**Multivariate**
	**Mean [95% CI]**	**Mean [95% CI]**	**Mean [95% CI]**	***R***^2^**; F (Df, residual);** ***p*****-value**	***R***^2^**; F (Df, residual);** ***p*****-value**	***R***^2^**; F (Df, residual);** ***p*****-value**	***R***^2^**; F (Df, residual);** ***p*****-value**
BMD_evol 1	−0.005 [−0.039, 0.030]	−0.008 [−0.036, 0.020]	0.0253 [−0.005, 0.056]	0.05; 0.988 (2, 37); 0.382	0.3; 1.15 (2, 32); 0.330	0.0; 0.14 (1, 38); 0.710	0.3; 0.17 (1, 33); 0.690
BMD_evol 2	0.0025 [−0.0196, 0.025]	0.024 [−0.0045, 0.053]	0.0319 [−0.0038, 0.0676]	0.06; 1.19 (2, 37); 0.317	0.2; 1.22 (2, 32); 0.310	0.1; 2.3 (1, 38); 0.138	0.2; 2.4 (1, 33); 0.130
BMD_evol 3	−0.0057 [−0.025, 0.0134]	0.0095 [−0.028, 0.0465]	0.0248 [−0.00148, 0.0511]	0.04; 0.89 (2, 37); 0.420	0.2; 0.92 (2, 32); 0.410	0.03; 1.26 (1, 38); 0.2690	0.2; 1.32 (1, 33); 0.260
T_evol 1	−0.060 [−0.360, 0.240]	−0.0722 [−0.332, 0.188]	0.300 [0.0345, 0.565]	0.05; 1.0 (2, 37); 0.380	0.25; 1.09 (2, 32) 0.350	0.0; 0.19 (1, 38); 0.670	0.2; 0.2 (1, 33); 0.650
T_evol 2	0.047 [−0.115, 0.209]	0.222 [0.0357, 0.409]	0.317 [0.010, 0.624]	0.07; 1.46 (2, 37); 0.250	0.33; 1.75 (2, 32); 0.190	0.07; 2.93 (1, 38); 0.095	0.33; 3.5 (1, 33); 0.070
T_ evol 3	−0.033 [−0.182, 0.116]	0.089 [−0.141, 0.319]	0.200 [−0.074, 0.474]	0.06; 1.15 (2, 37); 0.327	0.2; 1.23 (2, 32); 0.310	0.04; 1.74 (1, 38); 0.196	0.23; 1.88 (1, 33); 0.180

*CI, confidence interval; Df, degrees of freedom; BMD_evol 1, 2, and 3, bone mineral density (in g/cm^2^) at lumbar spine, right femoral neck, and proximal right hip, respectively; T_evol 1, 2, and 3, t-score values at lumbar spine, right femoral neck, and proximal right hip, respectively). R^2^ refers to the ratio of explained variation over total variation, varying between 0 and 1. F refers to the model mean square over the residual mean square. Residuals refers to the difference between the observed value of the outcome variable and the predicted value. P-value refers to probability of obtaining a result equal to or more extreme than the result that was obtained, assuming that the null hypothesis is true. The multivariate analyses involved adjustments for the quantitative covariable age, as well as the categorical covariables sex and the presence or absence of prednisone, bisphosphonate, and proton pump inhibitor treatment at 1-year post-transplantation*.

## Discussion

This study reports for the first time an association between rs1045642 genotypes in the *ABCB1* gene and blood pressure at 1-year post-transplantation among 40 KT recipients. We also observed a clinically relevant, yet not statistically significant, association between the rs1045642 polymorphism and the changes in BMD and t-scores at 1 year after KT.

Rs1045642 has been associated with hypertension and chronic kidney disease in the Chinese population, especially in the elderly (Liu et al., 2013). In a study involving 72 families of African descent, day and night ambulatory BPs were higher among patients with higher urinary excretion of sodium. Furthermore, a statistically significant negative interaction was found between *ABCB1* and *CYP3A5* polymorphisms and BP in the group with the highest urinary sodium excretion (Eap et al., 2007). Specifically, the T allele for the rs1045642 genotype and the CYP3A5^*^1 allele were both associated with a lower BP. In another study, involving 105 healthy volunteers, 137 patients with hypertension responding to treatment, and 83 with resistant hypertension, homozygous status for the C allele of the 3435CT polymorphism was associated with higher mean day and night SBP and DBP among patients with resistant hypertension (24-h BP measurement; *p* < 0.01; Lacchini et al., 2014).

These results are consistent with our findings, as KT recipients with a CC genotype tended to have a higher SBP and DBP 1-year post-transplantation. As prednisone is a well-established substrate of P-GP, coded by *ABCB1* (Dilger et al., 2004; Crowe and Tan, 2012), previous association studies have examined the response to prednisone therapy. For instance, the T allele for rs1045642 was associated with a delayed therapeutic response and steroid resistance among children with nephrotic syndrome treated with prednisone (Wasilewska et al., 2007; Jafar et al., 2011). These results indicate that reduced P-GP function due to the alternative mRNA splicing encountered with the rs1045642 polymorphism may lead to increased drug exposure and, therefore, increased side effects.

Regarding BMD changes, we observed stable BMD and t-score values among KT recipients homozygous for the C allele, whereas trends toward increased BMD and t-scores were observed in KT recipients with a CT or TT genotype. Interestingly, several genetic association studies and meta-analyses have reported an association between *ABCB1* polymorphisms and steroid-induced osteonecrosis (Gong et al., 2013; Li et al., 2014; Zhang et al., 2014, 2015; Zhou et al., 2015). These studies indicated that the T allele for rs1045642 was protective against the development of steroid-induced osteonecrosis.

The strengths of our study include our well-defined cohort. We were, thereby, able to evaluate two phenotypes (BP and bone mineralization). This study is the first report linking genetic polymorphism to the development of corticosteroid-related adverse events after KT. Prolonged use of corticosteroids after KT is still a matter of debate (Friedman, 2014; Thomusch et al., 2017). As illustrated by an updated review from the Cochrane Collaboration, strategies aimed at reducing or avoiding corticosteroids after KT increase the risk of rejection (Haller et al., 2016). However, data on the long-term effects of these strategies were lacking until the results from the Danish national registry compared data from renal transplantation centers using standard immunosuppression (Centers 2–4) to data from a steroid-free center (Center 1). The adjusted post-KT cancer risk was 6–39% lower in Centers 2–4 than in Center 1 (Engberg et al., 2016). Thus, establishing a strategy to identify patients who will benefit from corticosteroid reduction to avoid unnecessary exposure and subsequent side effects is of uttermost importance.

It is to be mentioned that rs1045642 polymorphism studies also report contradictory results with either inconclusive or opposite associations between TT genotypes and lowered P-gp function (Hodges et al., 2011). This lack of reproducibility may indicate discrepancies in the design or implementation of the studies themselves. For example, if a phenotype is not clearly defined or is too broadly defined, subjects may be misclassified. Power issue may result in the absence of impact, whereas phenotypes may be affected by a multitude of factors, including environmental and genetic elements, which may not be considered during study development. Environmental factors, such as previous xenobiotic exposure, are inherently difficult to control, but may significantly influence the phenotypes in question. However, in our single center retrospective pilot pharmacogenetics study, we addressed these issues by limiting our inclusion criteria to patients of Caucasian ethnicity having their first single organ kidney transplantation. In the exclusion criteria, we carefully considered that we wanted patients having a similar exposure to corticosteroids and we excluded patients receiving corticosteroids 12 months before (treatment of the primary renal disease) and 12 months after (treatment of rejection) kidney transplantation.

Among potential limitations, data concerning classes of antihypertensive drugs used before and at 1 year post transplantation were not collected. However, we were able to quantify the number of antihypertensive drugs used before and at 1 year post KT and did not observe a difference between genotypes and therefore, antihypertensive therapy is not expected to modify the association that we observed. The number of included patients may have been too low to achieve statistical significance for the BMD and t-score associations. Additionally, the effect of bisphosphonate use (70% of patients included in this study) is probably substantial and likely overshadowed any potential effects of rs1045642 on bone-associated outcomes. Our main outcome was a single office measurement of BP, and the absence of repeated measurements and/or 24-h ambulatory measurements prevents us from making broad conclusions.

In conclusion, this pilot study demonstrated that rs1045642 polymorphism may be associated with office SBP and DBP measurements at 1-year post-KT. The polymorphism also exhibited a trend toward being associated with changes in bone mineralization phenotypes at 1-year post-KT; this association was clinically, although not statistically, significant. These, preliminary results require confirmation in a larger independent cohort to further explore the potential use of rs1045642 determination in tailoring corticosteroid based-regimens for individual KT recipients to avoid the development of corticosteroid side effects.

## Author contributions

YB and KH: Designed research; YB and LS: Performed research; YB and BP: Collected data; YB, LS, BP, SF, and KH: Analyzed data; AP-G: Contributed important reagents; YB, LS, BP, AP-G, and KH: Wrote the paper.

### Conflict of interest statement

The authors declare that the research was conducted in the absence of any commercial or financial relationships that could be construed as a potential conflict of interest.

## Supplementary material

Supplemental Figure 1 Systolic and diastolic blood pressure measurement at 1 year after kidney transplantation and changes in bone mineral density (BMD) and t-score at 1-year post-transplantation according to rs1045642 genotype (CC, CT, TT).

## Abbreviations

ABCB1: ATP-binding cassette, sub-family B (MDR/TAP), member 1
BMD: bone mineral density
BP: blood pressure
DBP: diastolic blood pressure
PPI: proton pump inhibitor
SBP: systolic blood pressure.
