# Supplementary figures and images for: Recipient rs1045642 Polymorphism Is Associated With Office Blood Pressure at 1-Year Post Kidney Transplantation: A Single Center Pharmacogenetic Cohort Pilot Study
Source: Front Pharmacol. 2018 Mar 5;9:184. doi: 10.3389/fphar.2018.00184 (PMC5844966; doi:10.3389/fphar.2018.00184)

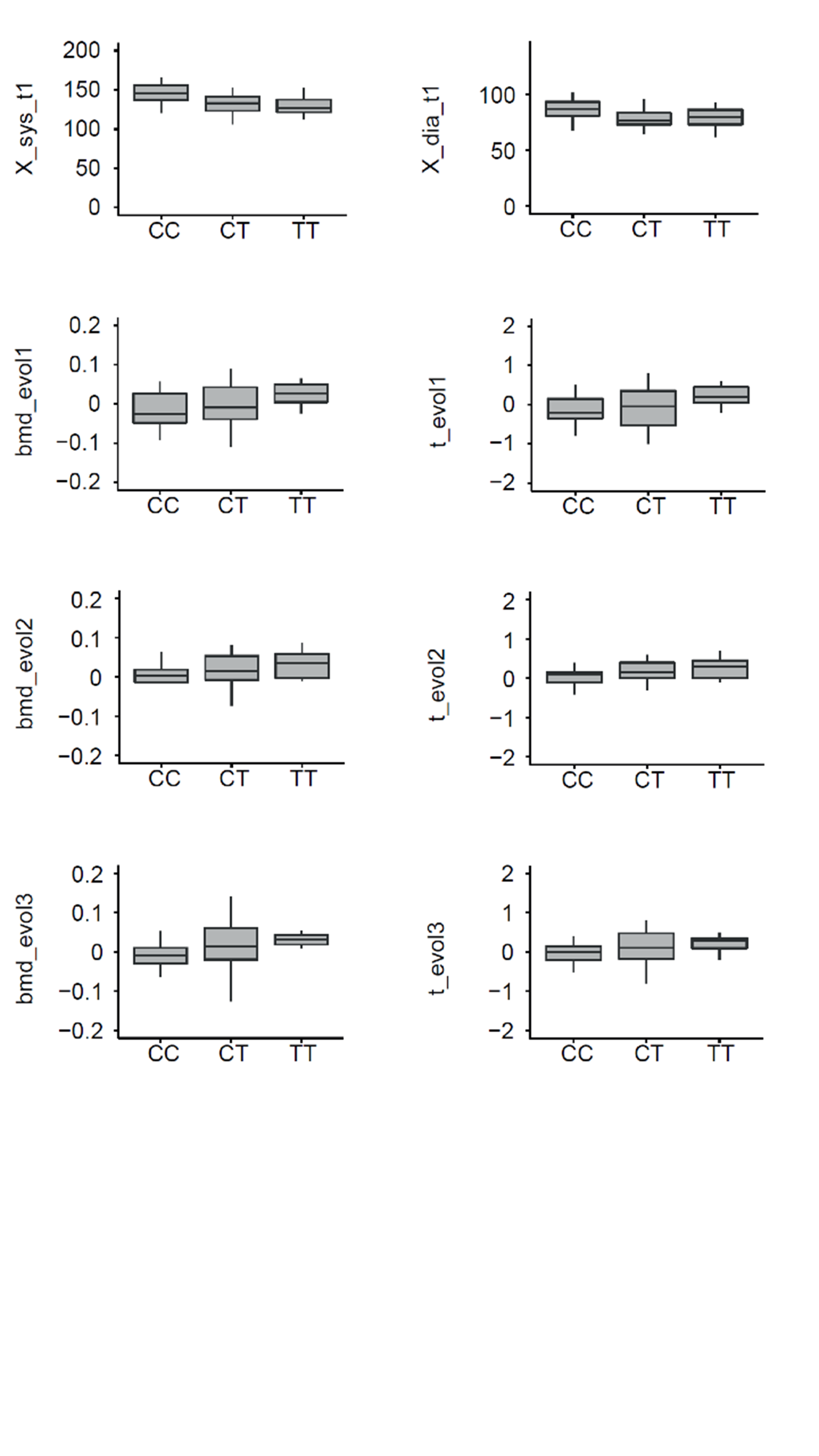

Supplement: Supplemental Figure 1 — Systolic and diastolic blood pressure measurement at 1 year after kidney transplantation and changes in bone mineral density (BMD) and t-score at 1-year post-transplantation according to rs1045642 genotype (CC, CT, TT). [file Image1.TIFF]
